# Who Is Pulling the Leash? Effects of Human Gender and Dog Sex on Human–Dog Dyads When Walking On-Leash

**DOI:** 10.3390/ani10101894

**Published:** 2020-10-16

**Authors:** Hao-Yu Shih, Mandy B. A. Paterson, Fillipe Georgiou, Nancy A. Pachana, Clive J. C. Phillips

**Affiliations:** 1 Centre for Animal Welfare and Ethics, University of Queensland, White House Building (8134), Gatton Campus, Gatton, QLD 4343, Australia; mpaterson@rspcaqld.org.au (M.B.A.P.); clive.phillips58@outlook.com (C.J.C.P.); 2 Royal Society for the Prevention of Cruelty to Animals Queensland, Brisbane, QLD 4076, Australia; 3 School of Mathematical and Physical Sciences, University of Newcastle, University Drive, Callaghan, NSW 2308, Australia; fillipegeorgiou@hotmail.com; 4 School of Psychology, University of Queensland, Brisbane, QLD 4072, Australia; n.pachana@psy.uq.edu.au

**Keywords:** gender, sex, on-leash walk, leash tension, behaviour, verbal cue, body gesture, human–dog interaction, shelter

## Abstract

**Simple Summary:**

The gender of humans and the sex of dogs influence human–dog interactions. This study investigated human–dog interactions when volunteers take shelter dogs for an on-leash walk, using video recording and a canine leash tension meter. Male dogs tended to pull more frequently and created higher leash tensions than female dogs. Dogs displayed more stress related behaviours when interacting with men than women, with the signs including spending less time holding the tail in a high position, and more frequent gazing and lip-licking behaviours. Finally, during the walk, there was a greater pre-disposition in women to use verbal commands and language typically associated with talking to babies, while men were more inclined to have physical contact with the dogs. These results may be used to match shelter dogs with appropriate men and women volunteers for dog walking exercise, and to improve potential socialisation of the dogs before rehoming.

**Abstract:**

Previous studies have indicated that human gender and canine sex influences human–dog interactions. However, the majority of studies have considered the interaction when dogs were off-leash and the behavioural interactions when dogs are walked on a leash have not been addressed. This study investigated human–dog interactions when shelter volunteers take shelter dogs for an on-leash walk. Video records were made of 370 walks, involving 74 volunteers and 111 shelter dogs, and a leash tension meter was used to determine the pull strength of dogs and walkers. Human gender and canine sex had dyadic effects during the walk. Male dogs tended to pull more frequently and created increased leash tensions. Dogs displayed more stress related behaviours when interacting with men than women, with the signs being spending less time holding the tail in the high position, and more frequent gazing and lip-licking behaviours. Finally, there was a greater pre-disposition in women to use verbal commands, and language typically used with babies, while men were more inclined to have physical contact with dogs. This study’s results may be used to match shelter dogs with appropriate men and women volunteers for walking exercise of the dog, and to improve potential dog socialisation efforts by shelters.

## 1. Introduction

Men and women interact with dogs in different ways, and dogs also respond to men and women differently [1,2,3]. Women tend to talk more and speak in an excited, high-pitched voice when interacting with dogs and they initiate talking to their dogs after a shorter latency [4]. In addition, women owners are more inclined to offer more encouraging behaviours to dogs perceived to be fearful [5]. These parental and caregiving behaviours are stronger and more developed in women and have been argued to be related to the human–dog attachment [4]. Men are more likely to use punishment-based rather than reward-based methods to train their dogs [6], and are more inclined to physically restrain their dogs [7]. Dogs for their part are able to discriminate between the human genders [8] and react differently toward men and women [2], in particular responding to men more with defensive-aggression as evidenced by increased barking and gazing [2].

The sex of dogs also affects their behaviour. Compared to their female counterparts, male dogs are bolder [9] and more likely to have behavioural issues, such as conspecific aggression, sexual problems and straying [10]. Male dogs are overrepresented in the behaviour clinic population, with greater risks of behaviour problems such as house soiling, coprophagia and destructive behaviour [11].

Previous research on human–dog interactions has mainly investigated behavioural interactions while dogs were off-leash [12,13]. Mandatory dog leash laws have been implemented in many countries around the world to protect wildlife [14], reduce disease transmission [15], prevent dog attacks and dog involvement in road traffic accidents [16,17]. There is a growing emphasis on the importance of loose leash heelwork, as a tense leash can be detrimental to the health of dogs by damaging their trachea and having negative effects on their cornea and intraocular pressure [18]. However, research about human–dog interactions when dogs are walked on a leash is limited.

Rein tension meters for horses have become popular in recent years among equestrians, enabling researchers and trainers to monitor how a rider communicates with the horse by measuring the force exerted on the reins by the human [19,20]. In this study, a similar concept was adopted for human–dog interactions. A custom-made leash tension meter was used to capture the leash tension when a dog was walked on a leash. Unlike the equine rein tension meter, our dog leash tension meter included an accelerometer in the device to differentiate between human and dog pulling during the walk [21].

Behavioural observation is another validated and common approach for assessing human–dog interactions [22,23]. Positions of their ears, tail and extremities, their facial expression, ongoing behaviours and general body tension are all important indicators of the dog’s stress levels [24,25]. In humans, behavioural observation is also variable, with body gestures and verbal cues being correlated with gender [4], personality [13] and training skills [26].

This study aimed to explore the role of human gender/dog sex in the behavioural interactions between volunteers and shelter dogs while dogs are walked on a leash, using a canine leash tension meter and video recorder. It was hypothesised first, that men volunteers walking male dogs would show highest leash tension. The second hypothesis was that shelter dogs would be more defensive and show more stress related behaviours when interacting with men volunteers [2], and thus would be more likely to look at the men volunteers and show lip-licking. Additionally, we hypothesised that women volunteers would talk to all shelter dogs, irrespective of sex, more frequently than do men volunteers, and tend to speak with a high-pitched voice. Finally, it was hypothesised that women volunteers would be more tolerant and satisfied with the interaction.

## 2. Materials and Methods

This study was approved by the Human Research Ethics and Animal Ethics Committees (Approval numbers: 2018001570 and SVS/400/18, respectively) of The University of Queensland.

### 2.1. Study Site

The research was conducted at the Royal Society for the Prevention of Cruelty to Animals, Queensland (RSPCA, QLD) shelter. Dogs were housed individually in rows of adjacent kennels (1.8 m wide × 1.2 m long × 3.0 m high) indoors and were able to make visual but not physical contact with one another across the central passage. Every enclosure was furnished with a metal crate, a raised mattress, a water bowl and enrichments (e.g., rubber toys or cardboard boxes). Each dog was walked twice daily, once in the morning from 08:00 to 10:00 h, and once in the afternoon from 14:00 to 16:00 h. The walk started from the kennel and ended with returning to the kennel, which took about 15 min to complete. In this study we only recorded the interaction when dogs were on the designated pathway, with dogs walked uni-directionally, so as not to be confounded by turning around. The time on the designated pathway was approximately 5 min. The ground of the designated pathway had several sections with different coverings to provide tactile and olfactory enrichment for the dogs. The first 40% was covered with gravel, followed by 20% on a concrete section, then 5% on wooden boards and finally the last 35% was covered with earth. Equipment and infrastructure provided for added stimulation and play including two bridges, two dog jumps, some tennis balls and some hanging plastic milk bottles.

### 2.2. Subjects

#### 2.2.1. Dogs

This study investigated 370 walks from July to early October 2019, involving 111 shelter dogs and 74 volunteers. All participating dogs had to have been resident at the RSPCA, Queensland, for at least one week, to enable them to become accustomed to the living and walking areas. Dogs’ walking behaviour was categorized into four levels by RSPCA animal attendants who had been closely working with those dogs. Levels were assigned according to the ease of walking the dogs, based on their performance during the daily walk. Level 1 dogs walked on a loose leash most of the time. Level 2 dogs pulled the leash during the walk occasionally and had more undesirable behaviours than level 1 dogs. Level 3 dogs tended to pull the leash fiercely due to excitement or timidity. Level 3+ dogs had severe behavioural issues, such as overt aggressiveness or fearfulness; however, they might or might not pull the leash harder than level 3 dogs. Dogs with severe behavioural or medical issues that might affect the observational study were excluded from the study because of safety and welfare concerns. All included dogs had undergone an RSPCA behavioural assessment [27].

#### 2.2.2. Volunteers

Volunteers were trained progressively in four stages, allowing them initially to walk dogs in level 1 and in each stage learning to walk the more challenging dogs. Volunteers could only walk level 1 dogs during their first month of volunteering; level 3+ volunteers were those who were most experienced and had gone through a series of standardized training programs. Volunteers could only walk dogs that had the same or lower level. Dogs were assigned to volunteers by experienced staff for a daily walk based on the volunteer’s training level.

### 2.3. Measures

#### Canine Leash Tension Meter

The custom designed canine leash tension meter (sampling rate: 10 Hz; measuring range: 0–100 kg-force; resolution: 100g-force) was commissioned for this project (RobacScience Pty Ltd, Blue Moutains, NSW, Australia) [21]. The device measured the force exerted on the leash and detected the direction of the pulling (handler versus dog). One end of the device had a metal handle to be held by the handler. The opposing end of the device had a stainless-steel eyebolt to allow a simple connection with a l.4-metre-long commercial dog leash (Rogz Snake Lead). A Windows 10 personal computer program was written for data collection and real-time displays. Recorded data were processed using MATLAB^®^ (MATLAB^®^ and Statistics Toolbox Release 2018b, The MathWorks, Inc., Natick, MA, USA) [21].

### 2.4. Research Design

Volunteers for this study were recruited via email, poster and direct recruitment by RSPCA staff and our researcher from the larger RSPCA QLD pool of dog-walking volunteers. Prize draws for ten $20 RSPCA World for Pets vouchers were offered as incentives. Since there were very few level 1 dogs during the research period, only volunteers with level 2 and above training levels were recruited. All participants had to have sufficient English proficiency to follow the research instructions and complete required documents. The research process was explained before the observational study and participants were informed on the research consent form that the overall study aim was to improve the interaction between volunteers and shelter dogs.

Dogs classified at the different levels were matched to volunteers of the right experience and training level by RSPCA staff. Dogs were walked on a designated pathway away from distractions at the shelter. Before every walk, the researcher held the leash tension meter vertically downward for 10 s when not connected to the dog. The signals generated were later used to help calibrate the recorded data using MATLAB. The volunteer attached the leash to both the collar and the harness in front of the dog’s chest [21], which provided a better control over the dog when it was lunging [28]. A laptop (Swift 3, Acer Inc., New Taipei City, Taiwan) was carried by the volunteer in a backpack for data collection, and a camera (GoPro Hero 7 Silver, GoPro^®^, San Mateo, CA, USA) was mounted on the volunteer’s head to record the walk and any interactions. Before walking, the volunteer was directed to pull the two ends of the device and hold the pull for 3 s by counting slowly “1, 2, 3”. This procedure was repeated three times in order to synchronize the tension data with the video. During the walk, the volunteer was instructed not to touch the leash unless the dog got tangled. The researcher also recorded the walk using an i-Phone 7 (Apple Inc., Cupertino, CA, USA) from 10 m behind.

### 2.5. Survey Instruments

Participants completed a consent form for the research, a demographic questionnaire and a personality test sealed in the envelopes on the desk at the RSPCA prior to the study if they received the research information through email/poster, or after the study if they were recruited by RSPCA staff and interacted with our researcher only in person on the day of their recorded walk. The personality test, NEO Five-Factor Inventory (NEO-FFI), measures human personality in five dimensions: neuroticism, extraversion, openness, conscientiousness and agreeableness [29]. At the end of each walk, participants completed the exit questionnaire (Table 1) containing 13 questions about their perspective of the walk they had just completed. For the exit questionnaire, there was initially a wide range of questions. To reduce the research time considering shelter operations, questions were reduced to the 13 most relevant questions. Two factors, human satisfaction factor and walkers’ perception of dog factor, were determined based on the exploratory factor analysis which was fully described in Table 2.

### 2.6. Ethograms

Canine behaviours (Table 3), human verbal cues (Table 4) and human body languages (Table 5) were coded using ethograms developed by referring to previous research [13,24,30,31,32,33] and modified during practice sessions. Behaviours were coded as ‘point events’ or ‘state events’. A point event indicates the number of times the event was observed and a state event was defined as the duration of the observed event.

### 2.7. Data Analysis

#### 2.7.1. Video Records of Dog and Human Behaviour

Three hundred and sixty-eight (*n* = 368/370) videos were coded in their entirety with Boris© behaviour observation software [34] using a continuous recording method. Two videos (*n* = 2/370) were unavailable due to technical problems. Videos were coded by the researcher who is a veterinarian and a certified dog trainer, who was trained in the use of ethograms and the software by two senior Ph.D. students. To blind the coder, video coding was completed prior to any analysis of human and canine demographics. Ten of the videos were chosen at random and double-coded to check intra-rater reliability. The average Cohen’s Kappa was 0.76.

#### 2.7.2. Leash Tension Analysis

Thirty-one (*n* = 31/370) leash tension files were lost, which we believe to be because the metal handle and case used to contain the components blocked the signal in a certain orientation [21], leaving 339 (*n* = 339/370) files for analysis.

Leash tension and pulling directions were calculated using MATLAB^®^ (MATLAB^®^ and Statistics Toolbox Release 2018b, The MathWorks, Inc., Natick, MA, USA). The start and end of each file were determined by matching the timestamps of video and the leash tension file, and also by matching three signal peaks at the beginning of the walk with the three repeated “1, 2, 3” verbal cues counted by the volunteer. Data were interpolated in order to make the sample times evenly spaced. Tension was tared by deducting the minimal value, which visually equals the baseline value when the device was not connected to the dog, from all measured values. Peak and average tension over the walk were calculated.

A ‘pull event’ was defined as a sharp peak of tension greater than the baseline tension, which corresponded to a sudden burst of pulling initiated by either the dog, the handler or both at the same time. A peak-finding algorithm with 0.1% of the body weight force set as a threshold was used to determine when ‘pull events’ occurred. An event started when the filtered tension exceeded the threshold and ended when either the filtered tension returned to below the threshold or the sign of the filtered tension gradient changed from negative to positive (indicating the start of a new pull event). Additionally, the directional signal of the accelerometer during the sample immediately prior to the start of a ‘pull event’ was used to determine the pulling direction [21].

Net maximal tension (NTmax), maximal tension by dog (DTmax) and handler (HTmax) were defined as the maximal tension throughout the walk, recorded for the dog and handler, respectively. Mean tension was calculated by averaging all tension peaks above the threshold. Net mean tension (NTmean), mean tension by dog (DTmean) and handler (HTmean) were defined as the mean tension throughout the walk, recorded for the dog and handler, respectively. Dog pulling frequency (DPF) and handler pulling frequency (HPF) were calculated by dividing the number of pulling events caused by the dog and the handler, respectively, by the total walking time [21].

### 2.8. Statistical Analysis

Statistical analysis was conducted using R version 3.6.1 [35] with packages leaps [36], MASS [37], car [38], carData [39], Matrix [40], polycor [41], plyr [42], psych [43], ggpubr [44] and nlme [45]. Personality scores of 5 dimensions were normally distributed based on observation; therefore, two sample t tests were used to compare the personality score between men and women.

To describe the exit questionnaire, exploratory factor analysis was performed with 13 questions, which revealed 2 factors. Negative question wording was deliberately used for one half of the questions; for these questions, reverse scores were used for the calculation of mean scores of the 2 factors. A human satisfaction factor (factor H, Cronbach’s alpha = 0.88) indicated the handler’s feeling about the walk. A higher factor H score indicated that the handler was more satisfied with the interaction. A walker’s perception of dog factor (factor D, Cronbach’s alpha = 0.83) represented the human’s perception of the dog. A higher factor D score indicated that the handler considered the dog better behaved, more supportive and being helped by the handler (see Table 1 and Table 2).

The 370 interactions were not independent because dogs were assigned to participants according to the training levels due to safety and welfare considerations. In addition, dogs that had been staying in the shelter longer during the research period tended to be walked more. Generalized linear mixed models were used for analysis to address the repeated measurements. To reduce the numbers of independent variables, a bivariate generalized linear model was used to analyse each combination of dependent (leash tension, pulling frequency, dog and human behaviour and exit questionnaire scores) and independent (human and dog demographics, human personality, canine behavioural assessment) variables. In the analysis of the exit questionnaire, apart from the above independent variables, canine and human behaviours, HTmax and HTmean were also entered as independent variables. Independent variables with *p*-values < 0.2 and those that were logically expected to influence the dependent variables, regardless of the *p*-value, were included in the generalized linear mixed model as fixed effects. Participants’ and dogs’ ID numbers were entered as random effects. Regression analysis started with a full model, in which all candidate variables were defined as predictors of interest. Independent variables with the highest *p*-values were then removed in a stepwise manner until the result of the model became consistent. In addition to assessing significance, the change in the Bayesian Information Criteria (BIC) was used to assess whether the model improved by entering or removing variables.

Data transformation (log and power transformation; see the footnotes of Table 6, Table 7, Table 8 and Table 9) was conducted on dependent variables for statistical analyses to meet the following assumptions of generalized linear mixed models: (1) residual normality (assessed by observation of quantile–quantile plots), (2) normality of the random effects (assessed by observation of quantile–quantile plots) and (3) homogeneity of variance of residuals (confirmed with either Levene’s Test or visual inspection of boxplots). The assumption of no collinearity between covariates was evaluated from variance inflation factors (VIF, ensuring that VIF < 2) [46].

This is the second report of a larger research project that explores the behavioural interaction between shelter dogs and volunteers during on-leash walks (see also [21]). This article reports on the effects of dog sex and human gender on the human–dog interactions during on-leash walks with respect to both human and dog behaviour. Other variables (e.g., human and canine demographics, personality and canine behavioural assessment) will be reported in future publications. Relationships between volunteers’ training levels and the leash tension/pulling frequency were reported in our previous article [21].

## 3. Results

### 3.1. Demographics

This study involved 111 shelter dogs and 74 human participants. For dogs, there were 58 (52.3%, *n* = 58/111) females and 53 (47.7%, *n* = 53/111) males, all gonadectomised. For human participants, there were 47 (63.5%, *n* = 47/74) women, 26 (35.1%, *n* = 26/74) men and 1 (1.4%, *n* = 1/74) person self-nominated as the third gender. Since there was only one third gender participant, to avoid potential bias, this person was excluded from all analysis regarding gender. Personality scores across five personality traits were not significantly different between men and women (Table 10).

### 3.2. Human Gender/Dog Sex and Leash Tension

Male dogs created higher maximal (*p* = 0.0011) and mean (*p* = 0.0026) tension and pulled more frequently (*p* = 0.0032), irrespective of the gender of the handler. Higher maximal (*p* = 0.0031) and mean (*p* = 0.0063) net tensions were observed when volunteers walked male dogs (Table 6). Handlers created higher mean tension (*p* = 0.0029) and higher pull frequency (*p* = 0.0018) when walking male dogs. Generally, human gender did not significantly influence the leash tension, except that dogs pulled less frequently (*p* = 0.0017) when being walked by men compared to women (Table 6). The above results partially support our hypothesis that male dogs are associated with higher leash tension; however, we did not find a correlation between human gender and leash tension.

### 3.3. Human Gender/Dog Sex and Canine Behaviour

A dog displayed less tracking behaviour when it was being walked by a man (*p* = 0.042); also tracking behaviour was less commonly observed in male dogs (*p* = 0.043). Dogs were less likely to keep their tail high (*p* = 0.023), but looked at the handler (*p* = 0.0013) and licked their own lips (*p* = 0.032) more frequently when walked by men. Finally, male dogs eliminated and marked more during the walk than female dogs (*p* = 0.0073) (Table 7). More gazing and lip-licking behaviours of dogs were observed when they were interacting with men, supporting the second hypothesis.

### 3.4. Human Gender/Dog Sex and Human Behaviour

Compared to women, men used fewer verbal cues in total (*p* = 0.0413) and, specifically, fewer high-pitched voices (*p* < 0.001) and commands (*p* = 0.0056) when interacting with or commanding the dog (Table 8). These findings support our third hypothesis that women tend to talk more and in higher pitched verbal expressions. However, men used more body language (*p* = 0.0089), specifically having more physical contacts (*p* = 0.0007) with the dogs during the walk (Table 9). Generally, the dog’s sex did not influence the handler’s verbal expression and body language. Finally, the items and the factor loadings obtained for the exit questionnaire are presented in Table 2. Results show that human gender and canine sex had no significant effects on the human experience of the walk.

## 4. Discussion

This study investigated the role of human gender and dog sex in the behavioural interactions when dogs are walked on a leash by different handlers. The first hypothesis was that a dog creates a tighter leash during the walk if it is male or it is walked by a man. We also hypothesised that dogs are more likely to show defensive and stress-related signs, such as staring and lip-licking if they are walked by men. Finally, it was hypothesised that women tend to speak to the dogs more often, commonly accompanied with a higher-pitched, baby-directed vocal communication and would be more tolerant and satisfied with the interaction.

### 4.1. Human Gender/Dog Sex and Leash Tension

Some support for the predicted associations between gender/sex and dyadic interaction was found. The first hypothesis that higher leash tension is observed among men and male dogs was partially met. Higher maximal and net tension were observed when male dogs were walked. Male dogs pulled harder and more frequently, making the handler pull harder and more frequently too. This finding may be explained by the fact that male dogs are more affected by testosterone, becoming more boisterous and aroused despite being gonadectomised [47,48,49]. Consequently, male dogs are generally bolder and showed a higher activity level than their female conspecifics [50,51,52], making them more likely to explore. It is also possible that male dogs are simply stronger than female dogs, as is the case in many other species [53]. Surprisingly, men handlers did not create higher tension, nor did they pull more frequently during the walk. Instead, when walking with men, the frequency of dog pulling was lower.

### 4.2. Human Gender/Dog Sex and Canine Behaviour

With respect to the dogs’ behaviour, fewer tracking behaviours, a shorter tail-high period and more frequent gazing and lip-licking behaviour were observed when the dogs were walked by men. This evidence supports the second hypothesis, indicating that dogs displayed more stress related signs and were more defensive towards the handler when interacting with men [54,55,56]. Additionally, such stress might decrease dogs’ interest in exploring the environment, contributing to a lower pulling frequency when walking with men. However, other studies have disagreed, indicating that sociability is positively associated with time dogs gazing at humans [57,58], and dogs that are perceived as more cooperative pull less on the leash and look more at their owners [59]. Nevertheless, considering other behaviours, including shorter tail-high period and more frequent lip-licking, it was more likely that in our study, rather than being more sociable, dogs were more stressed when walking with men. Dogs were often found to be more vigilant with men than women by gazing at the men more [2,54]. Physiologically, dogs have lower cortisol concentrations when interacting with women handlers, presumably because women are more likely to engage in tending and befriending behaviours [60] that attenuate dogs’ stress response [61]. Alternatively, it may be that men are considered more intimidating in terms of their body size, or dogs remember previous bad experiences of interacting with men [62].

The effects of canine sex on their behaviours were equally pronounced. In particular, male dogs spent less time tracking during the walk. A possible explanation may be that male dogs were better at identifying the direction of the scent; thereby male dogs tracked and located the target faster than their female counterparts [63]. Elimination and marking behaviours were also more commonly seen in male dogs, even though they were walked on a leash [64], which may also be influenced by testosterone despite orchiectomy having been performed [48]. However, in contrast to the previous finding that female dogs spent less time looking towards humans, our results revealed no difference regarding the gaze time between male and female dogs [2].

### 4.3. Human Gender/Dog Sex and Human Behaviour

The third hypothesis regarding the difference between how men and women talk to dogs was partially supported. Women used more verbal cues and commands and tended to talk to dogs using a high-pitched voice [4] even toward shelter dogs that shared weaker bonds with them. This finding may explain why dogs pulled more frequently when interacting with women, because women were more likely to excite the dog with frequent and exciting verbal expressions. The other assumption explaining the lower pulling frequency of dogs when walking with men, may be their tactile-friendly interaction style [1]. However, women have not been found to encourage dogs more by praising them [5]. This finding may be due to the fact that volunteers were required to walk each dog for a short period of time since there were many dogs to be walked. Consequently, instead of encouraging dogs, volunteers tended to ask them to “come”.

Men were not found to use more negative verbal cues but they were more likely to initiate physical contacts, which is in line with a previous study showing that men controlled their dogs more by physically restraining and holding them [7]. As a result, dogs become calmer as there are more frequent and longer physical contacts with men [31].

Nevertheless, we believe that our assumption of a high canine stress level causing a lower pulling frequency when walked by men, as previously described, is more plausible, because many stress indicators of dogs were observed (e.g., fewer tracking behaviours, a shorter tail-high period, more frequent gazing and lip-licking behaviour) [2,56,65]. Finally, it has been argued that women are more affected by dogs perceived to be fearful, making women present more encouraging behaviours [5]. However, in this study (in an animal shelter), the frequency of verbally praising dogs was not significantly different between men and women.

### 4.4. Human Gender/Dog Sex and Walking Experience

In this study, human gender and canine sex had no significant effects on the human experience of the walk. On average in past research, women score higher in neuroticism and agreeableness [66], whereas men are higher in extraversion [1]. In our limited sample, no significant difference between the genders in any of the personality traits was detected. We expect that with a larger sample one would indeed find a female bias with respect to neuroticism-related attachment and a male bias towards extraverts, who mainly appreciate their dogs as partners in shared activities.

### 4.5. Limitations

A limitation of our study was that the 370 interactions were not completely independent because the same volunteers and dogs participated in more than one walk, in order to create a larger sample size of walks. In addition, dogs were not randomly matched with participants, but were assigned to participants based on both the level assigned by shelter staff to dogs and participants. However, considering human safety, animal welfare and the operation of the shelter, the present results suggest potential ways to maximise the benefits and enjoyment of the walk for both dogs and people, by paying some attention to both the experience and gender of volunteers. Finally, a substantial limitation of this research was that the only coder of the data was familiar with the hypotheses of the study and was aware of handlers’ gender when coding videos. To prevent the potential bias, future studies should involve independent video coders naive to the study hypotheses.

## 5. Conclusions

This is the first study investigating the human–dog interactions during an on-leash walk focusing on shelter dogs and volunteers and the first time a leash tension meter was used for canine science. Results showed that male dogs generally caused higher leash tension and pulling frequency when walked on a leash. Dogs showed more stress-related signs when interacting with men; stress signs included shorter tail-high periods, more frequently looking toward men and lip-licking behaviour. Finally, there was a greater pre-disposition in women to use language as a relational tool, while men were more inclined to have physical contact with dogs. It is noted that the numeric differences (expressed in % or frequency) in both canine and human behaviours were small so the results should be interpreted with caution. However, the average walking time recorded in this study was only around 5 min. In reality, the entire time for each walk at the RSPCA would be 15–30 min so such differences (in time or counts of a behaviour) could be larger. This study may be useful in improving shelter procedures or decision-making processes about walking partners for dogs. For instance, for the safety concern, incoming male dogs without a previous behavioural history could be recommended to be initially walked by more experienced volunteers while the shelter gathers information on the dog’s behaviour, rather than less experienced volunteers who might have difficulties handling strong and sudden dog pulling. Compared to men, women may be better candidates to interact with fearful and stressed dogs. Finally, when interacting with dogs, especially timid individuals, men should be reminded to be more aware of any physical contacts that may escalate the stress-related behaviour of the dog.

## Figures and Tables

**Table 1:** Exit questionnaire for volunteers (*n* = 74) following walking dogs (*n* = 111) on a designated route at Royal Society for the Prevention of Cruelty to Animals (RSPCA) Queensland, requiring them to rate each sentence on a 5-point scale from 1 (strongly disagree) to 5 (strongly agree).

1. The dog’s behaviour was good.
2. I could not handle the dog well.
3. I felt comfortable when interacting with the dog.
4. I was physically tense.
5. Overall, this is a good experience.
6. The interaction was challenging for me.
7. The dog did not understand me well.
8. I did not feel that I was helping the dog.
9. I felt supported by the dog.
10. I did not enjoy its company.
11. I would love to walk this dog again on another day.
12. I don’t think this dog is suitable for a non-experienced adopter.
13. I think the dog is ready for adoption.

Human satisfaction factor (Factor H) utilised responses to questions 2, 3, 4, 5, 6, 10, 11. Walker’s perception of dog factor (Factor D) utilised responses to questions 1, 7, 8, 9, 12, 13 (see Table 2).

**Table 2:** Factor loadings for the 13 items in the exit questionnaire.

	Factor H	Factor D
1.	0.537	**−0.581**
2.	**−0.556**	0.443
3.	**0.694**	−0.325
4.	**−0.680**	0.316
5.	**0.739**	-0.331
6.	**−0.559**	0.419
7.	-0.253	**0.689**
8.	-0.353	**0.518**
9.	0.318	**−0.644**
10.	**−0.503**	0.287
11.	**0.631**	-0.387
12.	−0.276	**0.456**
13.	0.478	**−0.475**

Loadings in bold indicate the factor upon which each item was selected. Human satisfaction factor (Factor H, Cronbach’s alpha = 0.88): 2, 3, 4, 5, 6, 10, 11. Walker’s perception of dog factor (Factor D, Cronbach’s alpha = 0.83): 1, 7, 8, 9, 12, 13.

**Table 3:** Ethogram of canine behaviour.

Behaviour	Description	Behaviour Type	Reference
Track	Dog moves along the ground with head lowered, using nose to follow a scent.	State event	[30]
Sniff	Dog orientates nose to within 5 cm of an object, wall or ground to explore or to express stress or appeasement.	State event	[30]
Eliminate-mark	Dog defecates or urinates in sitting, squatting or standing position	Point event	[24]
Shake	Dog shakes its body or head.	Point event	
Pant	Dog keeps its mouth wide open and breathes vigorously.	State event	[30]
Gaze	Dog looks toward the handler.	Point event	[30]
Lip-lick	Part of tongue is shown and moved along the upper lip or snout.	Point event	[30]
Tail wag	Tail is moving from side to side.	State event	[31]
Tail high	Tail is held stiffly and upright, either curled over the back or straight.	State event	[32]

Point event: the number of times the event was observed. State event: the duration of the observed event.

**Table 4:** Ethogram of human verbal cues.

Behaviour	Description	Behaviour Type	Reference
Sit	Volunteer asks the dog to sit.	Point event	
Command	Volunteer talks to the dog with an utterance containing a single command (e.g., “Stay!” “Come!” “Let’s go!”).	Point event	[13]
Attention seeking	Volunteer tries to get the attention of the dog and calls the dog by its name and/or the utterance of “Look!”, and/or clicking the tongue (“tze tze” sound).	Point event	[13]
High-pitched voice	Volunteer talks to the dog with high pitched voice with baby-talk expressions.	Point event	[31]
Praise	Volunteer talks to the dog with a positive utterance (e.g., “Great!” “Well done!” “Good dog!”).	Point event	[13,31]
Negative verbal cue	Volunteer talks to the dog with a negative utterance (e.g., “No!” “Bad dog!” “Don’t …” “Stop chewing the leash” “Let the leash (it) go”).	Point event	
Communication	Volunteer tries to communicate with the dog or to ask the dog some questions. (e.g., “Which way do you want to go?” “What are you sniffing at?” “Do you want to fetch?” “Do you want to drink?”)	Point event	[33]

Point event: the number of times the event was observed. State event: the duration of the observed event.

**Table 5:** Ethogram of human body language.

Behaviour	Description	Behaviour Type	Reference
Gestural	Volunteer displays voluntary hand movement directed towards the dog (e.g., referential point, patting his/her own thigh, luring the dog with a hand or food).	Point event	[13,31]
Physical contacts	Physical contacts initiated by the volunteer, including contacts when treats were given.	Point event	
Treat	Food is given to the dog.	Point event	

Point event: the number of times the event was observed.

**Table 6:** Generalized linear mixed model of dog sex and human gender effects on leash tension and pulling frequency. Mean (kg force) (μ) and standard deviation (SD) of different dependent variables by gender/sex are provided, together with β, SE and *p*-values for significant or close to significant variables.

	Log_10_NT_max_	Log_10_NT_mean_	Log_10_DT_max_	Log_10_DT_mean_	Log_10_DPF	Log_10_HT_max_	Log_10_HT_mean_	Log_10_HPF
**Human** **Gender ^1^**	Women	Women	Women	Women	Women	Women	Women	Women
	μ 3.72, SD 2.01	μ 0.58, SD 0.26	μ 3.24, SD 1.81	μ 1.15, SD 0.5	μ 0.19, SD 0.14	μ 3.05, SD 1.72	μ 1.14, SD 0.49	μ 0.19, SD 0.13
	Men	Men	Men	Men	Men	Men	Men	Men
	μ 3.68, SD 1.93	μ 0.59, SD 0.24	μ 3.29, SD 1.87	μ 1.16, SD 0.48	μ 0.18, SD 0.13	μ 2.97, SD 1.77	μ 1.14, SD 0.52	μ 0.17, SD 0.12
	--	--	--	--	*β* -0.29 SE 0.09 *p* 0.0017	--	--	--
**Dog** **Sex ^1^**	Female	Female	Female	Female	Female	Female	Female	Female
	μ 3.3, SD 1.72	μ 0.54, SD 0.22	μ 2.88, SD 1.58	μ 1.07, SD 0.39	μ 0.17, SD 0.13	μ 2.72, SD 1.5	μ 1.03, SD 0.38	μ 0.16, SD 0.1
	Male	Male	Male	Male	Male	Male	Male	Male
	μ 4.12, SD 2.13	μ 0.63, SD 0.27	μ 3.64, SD 1.97	μ 1.25, SD 0.56	μ 0.21, SD 0.14	μ 3.35, SD 1.9	μ 1.24, SD 0.58	μ 0.21, SD 0.14
	*β* 0.22	*β* 0.15	*β* 0.26	*β* 0.17	*β* 0.37	*β* 0.15	*β* 0.168	*β* 0.36
	SE 0.071	SE 0.053	SE 0.078	SE 0.054	SE 0.12	SE 0.076	SE 0.055	SE 0.11
	*p* 0.0031	*p* 0.0063	*p* 0.0011	*p* 0.0026	*p* 0.0032	*p* 0.051	*p* 0.0029	*p* 0.0018

Tension and pulling frequency were analysed after log10 transformation. NTmax: maximal net leash tension. NTmean: mean net leash tension. DTmax: maximal leash tension caused by dog. DTmean: mean leash tension caused by dog. HTmax: maximal leash tension caused by handler. HTmean: mean leash tension caused by handler. DPF: dog pulling frequency. HPF: handler pulling frequency. μ: mean (kg force). SD: standard deviation of μ. *β*: regression coefficient. SE: standard error of *β*. *p*: *p*-value of the model. --: Not included in the generalized linear mixed model because the independent variable had high *p*-values in the bivariate regression model. ^1.^ Women and female dogs were used as control.

**Table 7:** Generalized linear mixed model of dog sex and human gender effects on canine behaviour. Mean (μ) and standard deviation (SD) of different dependent variables by gender/sex was provided.

	Track (%)	Tail High (%)	Gaze (no./s)	Lip-Lick (no./s)	Eliminate-Mark (no./s) ^3^	Pant (%)
**Human Gender ^1^**	Women	Women	Women	Women	Women	Women
	μ 15.51, SD 11.62	μ 78.59, SD 29.85	μ 0.01, SD 0.02	μ 0.01, SD 0.01	μ 0.01, SD 0.01	μ 10.48, SD 11.07
	Men	Men	Men	Men	Men	Men
	μ 14.64, SD 10.57	μ 76.82, SD 30.43	μ 0.02, SD 0.02	μ 0.01, SD 0.02	μ 0.01, SD 0.01	μ 10.36, SD 11.2
	*β* −0.027	*β* −0.073	*β* 0.037	*β* 0.024	--	--
	SE 0.013	SE 0.032	SE 0.012	SE 0.011		
	*p* 0.042	*p* 0.023	*p* 0.0013	*p* 0.032		
**Dog Sex ^1^**	Female	Female	Female	Female	Female	Female
	μ 17.18, SD 12.58	μ 72.32, SD 33.04	μ 0.01, SD 0.02	μ 0.01, SD 0.02	μ < 0.01, SD < 0.01	μ 8.31, SD 10.4
	Male	Male	Male	Male	Male	Male
	μ 12.82, SD 9.16	μ 84.42, SD 24.84	μ 0.02, SD 0.02	μ 0.01, SD 0.01	μ 0.01, SD 0.01	μ 12.48, SD 11.38
	*β* −0.041	*β* 0.038	*β* −0.0033	--	*β* 0.013	*β* −0.024
	SE 0.02	SE 0.06	SE 0.016		SE 0.0049	SE 0.029
	*p* 0.043	*p* 0.53	*p* 0.84		*p* 0.0073	*p* 0.41

Track (%): tracking time (s)/total walking time (s) × 100%. Tail high (%): tail high time (s)/total walking time (s) × 100%, analysed in power of 7. Gaze (no./s): Numbers of gazes / time when the dog’s head was visible in the Gopro video (s), analysed in power of 0.4. Lip-lick (no./s): Numbers of lip-licks/time when the dog’s head was visible in the Gopro video (s), analysed in power of 0.4. Eliminate-mark (no./s): Numbers of eliminate-marks/total walking time (s), analysed in power of 0.6. Pant (%): painting time (s)/time when the dog’s head was visible in the Gopro video (s) × 100%, analysed in power of 0.5. μ: mean. SD: standard deviation of μ. *β*: regression coefficient. SE: standard error of *β*. *p*: *p*-value of the model. --: Not included in the generalized linear mixed model because the independent variable had high *p*-values in the bivariate regression model. ^1^ Women and female dogs were used as control. Wagging tail, shaking body and sniffing were not entered into the generalized linear mixed model because both independent variables, dog sexes and human genders, had high *p*-values in the bivariate regression models.

**Table 8:** Generalized linear mixed model of dog sex and human gender (independent variables) effects on human verbal cues (dependent variables). Mean (μ) and standard deviation (SD) of different dependent variables by gender/sex was provided. All verbal cues were analysed as frequencies (numbers of events/total walking time).

	Total Verbal Cues (no./s) ^1^	Attention Seeking (no./s) ^2^	Communication (no./s) ^2^	Negative Verbal Cue (no./s) ^2^	Praise (no./s) ^1^	High-Pitched Voice (no./s) ^1^	Command (no./s) ^1^
**Human Gender ^3^**	Women	Women	Women	Women	Women	Women	Women
	μ 0.09, SD 0.07	μ 0.02, SD 0.02	μ 0.01, SD 0.01	μ < 0.01, SD 0.01	μ 0.02, SD 0.02	μ 0.02, SD 0.02	μ 0.03, SD 0.03
	Men	Men	Men	Men	Men	Men	Men
	μ 0.07, SD 0.06	μ 0.02, SD 0.02	μ < 0.01, SD < 0.01	μ < 0.01, SD < 0.01	μ 0.02, SD 0.03	μ < 0.01, SD 0.01	μ 0.03, SD 0.02
	*β* −0.034	*β* −0.031	*β* −0.003	*β* −0.011	*β* 0.005	*β* −0.062	*β* −0.032
	SE 0.017	SE 0.017	SE 0.012	SE 0.0081	SE 0.011	SE 0.011	SE 0.011
	*p* 0.041	*p* 0.076	*p* 0.8	*p* 0.17	*p* 0.65	*p* < 0.001	*p* 0.0056
**Dog Sex ^3^**	Female	Female	Female	Female	Female	Female	Female
	μ 0.08, SD 0.06	μ 0.02, SD 0.02	μ 0.01, SD 0.01	μ < 0.01, SD < 0.01	μ 0.02, SD 0.02	μ 0.01, SD 0.02	μ 0.03, SD 0.03
	Male	Male	Male	Male	Male	Male	Male
	μ 0.08, SD 0.07	μ 0.02, SD 0.02	μ < 0.01, SD 0.01	μ < 0.01, SD < 0.01	μ 0.02, SD 0.03	μ 0.01, SD 0.02	μ 0.03, SD 0.03
	--	--	--	--	--	--	*β* −0.021 SE 0.012 *p* 0.085

^1.^ Analysed after transformation to the power of 0.5. ^2.^ Analysed after transformation to the power of 0.4. ^3^ Women and female dogs were used as control. μ: mean. SD: standard deviation of μ. *β*: regression coefficient. SE: standard error of *β*. *p*: *p*-value of the model --: Not included in the generalized linear mixed model because the independent variable had high *p*-values in the bivariate regression model.

**Table 9:** Generalized linear mixed model of dog sex and human gender (independent variables) effects on human body language (dependent variables). Mean (μ) and standard deviation (SD) of different dependent variables by gender/sex was provided. All body languages were analysed as frequencies (numbers of events/total walking time).

	Total Body Language (no./s) ^1^	Treating Dog with Food (no./s)	Physical Contacts (no./s) ^1^
**Human Gender ^2^**	Women	Women	Women
	μ 0.01, SD 0.02	μ < 0.01, SD < 0.01	μ < 0.01, SD 0.01
	Men	Men	Men
	μ 0.01, SD 0.02	μ < 0.01, SD 0.01	μ < 0.01, SD 0.01
	*β* 0.06	*β* 0.001	*β* 0.067
	SE 0.023	SE 0.00063	SE 0.019
	*p* 0.0089	*p* 0.11	*p* 0.0007
**Dog Sex ^2^**	Female	Female	Female
	μ 0.01, SD 0.02	μ < 0.01, SD 0.01	μ < 0.01, SD 0.01
	Male	Male	Male
	μ 0.01, SD 0.02	μ < 0.01, SD <0.01	μ < 0.01, SD 0.01
	--	--	--

^1.^ Analysed after transformation to the power of 0.3. ^2.^ Women and female dogs were used as control. μ: mean. SD: standard deviation of μ. *β*: regression coefficient. SE: standard error of *β*. *p*: *p*-value of the model. --: Not included in the generalized linear mixed model because the independent variable had high *p*-values in the bivariate regression model. Body gestures and asking the dog to sit were not entered into the generalized linear mixed model because both independent variables, dog sexes and human genders, had high *p*-values in the bivariate regression models.

**Table 10:** Comparison of mean personality scores (μ) and stand deviation (SD) between men and women volunteers across five personality traits (Two sample *t*-test).

Neuroticism	Extraversion	Openness	Conscientiousness	Agreeableness
Women: μ 25.64, SD 9.1	Women: μ 27.11, SD 7.27	Women: μ 28.98, SD 5.99	Women: μ 30.96, SD 6.6	Women: μ 35.28, SD 6.07
Men: μ 23.65, SD 8.26	Men: μ 27.73, SD 8.66	Men: μ 30.81, SD 7.07	Men: μ 30.04, SD 8.36	Men: μ 32.81, SD 5.55
*p*-value = 0.35	*p*-value = 0.76	*p*-value = 0.28	*p*-value = 0.64	*p*-value = 0.09

μ: mean. SD: standard deviation of μ.

## References

[1] Kotrschal K., Schoberl I., Bauer B., Thibeaut A.-M., Wedl M. (2009). Dyadic relationships and operational performance of male and female owners and their male dogs. Behav. Process..

[2] Wells D.L., Hepper P.G. (1999). Male and female dogs respond differently to men and women. Appl. Anim. Behav. Sci..

[3] McGuire B., Fry K., Orantes D., Underkofler L., Parry S. (2020). Sex of Walker Influences Scent-marking Behavior of Shelter Dogs. Animals.

[4] Prato-Previde E., Fallani G., Valsecchi P. (2006). Gender Differences in Owners Interacting with Pet Dogs: An Observational Study. Ethology.

[5] Pirrone F., Pierantoni L., Mazzola S.M., Vigo D., Albertini M. (2015). Owner and animal factors predict the incidence of, and owner reaction toward, problematic behaviors in companion dogs. J. Vet. Behav..

[6] Blackwell E.J., Bolster C., Richards G., Loftus B.A., Casey R.A. (2012). The use of electronic collars for training domestic dogs: Estimated prevalence, reasons and risk factors for use, and owner perceived success as compared to other training methods. BMC Vet. Res..

[7] Aliabadi I., Wedl M., Schoberl I., Bauer B., Kotrschal K. Effects of gender on performance in human-dog dyads in an agility parcours. Proceedings of the 2010 Canine Science Forum.

[8] Ratcliffe V.F., McComb K., Reby D. (2014). Cross-modal discrimination of human gender by domestic dogs. Anim. Behav..

[9] Starling M.J., Branson N., Thomson P.C., McGreevy P.D. (2013). Age, sex and reproductive status affect boldness in dogs. Vet. J..

[10] Wells D.L., Hepper P.G. (2000). Prevalence of behaviour problems reported by owners of dogs purchased from an animal rescue shelter. Appl. Anim. Behav. Sci..

[11] Col R., Day C., Phillips C.J.C. (2016). An epidemiological analysis of dog behavior problems presented to an Australian behavior clinic, with associated risk factors. J. Vet. Behav..

[12] Rooney N.J., Bradshaw J.W.S., Robinson I.H. (2000). A comparison of dog–dog and dog–human play behaviour. Appl. Anim. Behav. Sci..

[13] Kis A., Turcsán B., Miklósi Á, Gácsi M. (2012). The effect of the owner’s personality on the behaviour of owner-dog dyads. Interact. Stud..

[14] Bowes M., Keller P., Rollins R., Gifford R. (2017). The Effect of Ambivalence on On-Leash Dog Walking Compliance Behavior in Parks and Protected Areas. J. Park Recreat. Adm..

[15] Day M.J., Breitschwerdt E., Cleaveland S., Karkare U., Khanna C., Kirpensteijn J., Kuiken T., Lappin M.R., McQuiston J., Mumford E. (2012). Surveillance of Zoonotic Infectious Disease Transmitted by Small Companion Animals. Emerg. Infect. Dis..

[16] Thompson P.G. (1997). The public health impact of dog attacks in a major Australian city. Med. J. Aust..

[17] Klainbart S., Bibring U., Strich D., Chai O., Bdolah-Abram T., Aroch I., Kelmer E. (2018). Retrospective evaluation of 140 dogs involved in road traffic accidents. Vet. Rec..

[18] Pauli A.M., Bentley E., Diehl K.A., Miller P.E. (2006). Effects of the Application of Neck Pressure by a Collar or Harness on Intraocular Pressure in Dogs. J. Am. Anim. Hosp. Assoc..

[19] Hawson L.A., Salvin H.E., McLean A.N., McGreevy P.D. (2014). Riders’ application of rein tension for walk-to-halt transitions on a model horse. J. Vet. Behav..

[20] Warren-Smith A.K., Curtis R.A., Greetham L., McGreevy P.D. (2007). Rein contact between horse and handler duringspecific equitation movements. Appl. Anim. Behav. Sci..

[21] Shih H.-Y., Georgiou F., Curtis R.A., Paterson M.B.A., Phillips C.J.C. (2020). Behavioural evaluation of a leash tension meter which measures pull direction and force during human-dog on-leash walks. Animals.

[22] Protopopova A., David C., Wynne L. (2014). Adopter-dog interactions at the shelter: Behavioral and contextual predictors of adoption. Appl. Anim. Behav. Sci..

[23] Foyer P., Svedberg A.-M., Nilsson E., Wilsson E., Faresjö A., Jensen P. (2016). Behavior and cortisol responses of dogs evaluated in a standardized temperament test for military working dogs. J. Vet. Behav..

[24] Palestrini C., Minero M., Cannas S., Rossi E., Frank D. (2010). Video analysis of dogs with separation-related behaviors. Appl. Anim. Behav. Sci..

[25] Siniscalchi M., Lusito R., Vallortigara G., Quaranta A. (2013). Seeing Left- or Right-Asymmetric Tail Wagging Produces Different Emotional Responses in Dogs. Curr. Biol..

[26] Alexandera M.B., Frienda T., Haug L. (2011). Obedience training effects on search dog performance. Appl. Anim. Behav. Sci..

[27] Clay L., Paterson M., Bennett P., Perry G., Phillips C. (2019). Early Recognition of Behaviour Problems in Shelter Dogs by Monitoring them in their Kennels after Admission to a Shelter. Animals.

[28] Royal Society for the Prevention of Cruelty to Animals South Australia The Best-Ever Walking Harness for Your Dog (and the Must-Avoid Collars and Leads). https://www.rspcasa.org.au/best-walking-harness-dogs/.

[29] McCrae R.R., Costa P.T. (2007). Brief Versions of the NEO-PI-3. J. Individ. Differ..

[30] Grainger J., Wills A.P., Montrose V.T. (2016). The behavioral effects of walking on a collar and harness in domestic dogs (Canis familiaris). J. Vet. Behav..

[31] McGowan R.T.S., Bolte C., Barnett H.R., Perez-Camargo G., François M. (2018). Can you spare 15 min? The measurable positive impact of a 15-min petting session on shelter dog well-being. Appl. Anim. Behav. Sci..

[32] Beerda B., Schilder M.B.H., van Hooff J.A.R.A.M., de Vries H.W., Mol J.A. (1998). Behavioural, saliva cortisol and heart rate responses to different types of stimuli in dogs. Appl. Anim. Behav. Sci..

[33] Cimarelli G., Turcsán B., Bánlaki Z., Range F., Virányi Z. (2016). Dog Owners’ Interaction Styles: Their Components and Associations with Reactions of Pet Dogs to a Social Threat. Front. Psychol..

[34] Friard O., Gamba M. (2016). BORIS: A free, versatile open-source event-logging software for video/audio coding and live observations. Methods Ecol. Evol..

[35] R Core Team (2019). A Language and Environment for Statistical Computing.

[36] Lumley T. (2020). Leaps: Regression Subset Selection.

[37] Venables W.N., Ripley B.D. (2002). Modern Applied Statistics with S.

[38] Fox J., Weisberg S. (2019). An {R} Companion to Applied Regression.

[39] Fox J., Weisberg S., Price B. (2020). CarData: Companion to Applied Regression Data Sets.

[40] Bates D., Maechler M. (2019). Matrix: Sparse and Dense Matrix Classes and Methods.

[41] Fox J. (2019). Polycor: Polychoric and Polyserial Correlations.

[42] Wickham H. (2011). The Split-Apply-Combine Strategy for Data Analysis. J. Stat. Softw..

[43] Revelle W. (2020). Psych: Procedures for Psychological, Psychometric, and Personality Research.

[44] Kassambara A. (2020). Ggpubr: ’ggplot2’ Based Publication Ready Plots.

[45] Pinheiro J., Bates D., DebRoy S., Sarkar D., R Core Team (2020). Nlme: Linear and Nonlinear Mixed Effects Models.

[46] Zuur A.F., Ieno E.N., Elphick C.S. (2010). A protocol for data exploration to avoid common statistical problems. Methods Ecol. Evol..

[47] Maarschalkerweerd R.J., Endenburg N., Kirpensteijn J., Knol B.W. (1997). Influence of orchiectomy on canine behaviour. Vet. Rec..

[48] Hart B.L., Eckstein A.R. (1997). The role of gonadal hormones in the occurrence of objectionable behaviours in dogs and cats. Appl. Anim. Behav. Sci..

[49] Warnes C. (2014). Five myths commonly associated with neutering in dogs. Vet. Nurs..

[50] Kubinyi E., Turcsán B., Miklósi Á (2009). Dog and owner demographic characteristics and dog personality trait associations. Behav. Process..

[51] Starling M.J., Branson N., Thomson P.C., McGreevy P.D. (2013). “Boldness” in the domestic dog differs among breeds and breed groups. Behav. Process..

[52] Hart B.L., Hart L.A., Serpell J. (2016). Breed and gender differences in dog behavior. The Domestic Dog: Its Evolution, Behavior and Interactions with People.

[53] Perez-Gomez J., Rodriguez G.V., Ara I., Olmedillas H., Chavarren J., González-Henriquez J.J., Dorado C., Calbet J.A.L. (2008). Role of muscle mass on sprint performance: Gender differences?. Eur. J. Appl. Physiol..

[54] Yong M.H., Ruffman T. (2015). Domestic dogs match human male voices to faces, but not for females. Behaviour.

[55] Leaver S.D.A., Reimchen T.E. (2008). Behavioural responses of Canis familiaris to different tail lengths of a remotely-controlled life-size dog replica. Behaviour.

[56] Tami G., Gallagher A. (2009). Description of the behaviour of domestic dog (Canis familiaris) by experienced and inexperienced people. Appl. Anim. Behav. Sci..

[57] Jakovcevic A., Mustaca A., Bentosela M. (2012). Do more sociable dogs gaze longer to the human face than less sociable ones?. Behav. Process..

[58] Bentosela M., Wynne C.D.L., D’Orazio M., Elgier A., Udell M.A.R. (2016). Sociability and gazing toward humans in dogs and wolves: Simple behaviors with broad implications. J. Exp. Anal. Behav..

[59] Roth L.S.V., Jensen P. (2015). Assessing companion dog behavior in a social setting. J. Vet. Behav..

[60] Buttner A.P., Thompson B., Strasser R., Santo J. (2015). Evidence for a synchronization of hormonal states between humans and dogs during competition. Physiol. Behav..

[61] Jones A.C., Josephs R.A. (2006). Interspecies hormonal interactions between man and the domestic dog (Canis familiaris). Horm. Behav..

[62] Herzog H.A. (2007). Gender Differences in Human–Animal Interactions: A Review. Anthrozoös.

[63] Wells D.L., Hepper P.G. (2003). Directional tracking in the domestic dog, Canis familiaris. Appl. Anim. Behav. Sci..

[64] Řezáč P., Viziová P., Dobešová M., Havlíček Z., Pospíšilová D. (2011). Factors affecting dog–dog interactions on walks with their owners. Appl. Anim. Behav. Sci..

[65] Firnkes A., Bartels A., Bidoli E., Erhard M. (2017). Appeasement signals used by dogs during dog-human communication. J. Vet. Behav..

[66] Weisberg Y.J., DeYoung C.G., Hirsh J.B. (2011). Gender differences in personality across the ten aspects of the Big Five. Front. Psychol..

